# Key genes of Taohong Siwu decoction in treating coronary heart disease based on network pharmacology and Mendelian randomization

**DOI:** 10.1097/MD.0000000000046856

**Published:** 2026-01-02

**Authors:** Chunbin Wang, Xin Li, Jian Wang

**Affiliations:** a Department of Cardiology, The Affiliated Hospital of Southwest Jiaotong University, Chengdu, China; b Department of Cardiology, The Third People’s Hospital of Chengdu, Chengdu, China.

**Keywords:** coronary heart disease, key genes, Mendelian randomization, Taohong Siwu decoction

## Abstract

It has been found in previous studies that Taohong Siwu Decoction (THSWD) has therapeutic effects on coronary heart disease (CHD). The aim of this study was to identify the related key genes of THSWD for the treatment of CHD and to examine the potential biological mechanisms of the key genes in CHD. The CHD-related genes (CHDRGs) were obtained from public databases, along with the bioactive components of THSWD and their corresponding drug-target genes. Candidate genes were identified by obtaining the intersection of CHDRGs and drug-target genes. Subsequently, Mendelian randomization analysis was utilized to determine the candidate key genes causally associated with CHD. The final key genes for CHD were obtained through Bayesian colocalization analysis. Following that, an active ingredient-target-pathway network was constructed based on the key genes to explore the interactions. Finally, molecular docking was conducted to verify the binding ability among the key genes and the bioactive components of the drug. In this study, a sum of 117 candidate genes was obtained by intersecting 547 CHDRGs with 880 drug-target genes. Subsequently, Mendelian randomization analysis was conducted to screen out 5 hazardous exposure factors as candidate key genes, namely ERBB2, PTPN22, FLT1, MPO, and signal transducer and activator of transcription 1 (STAT1). Bayesian analysis was then carried out, which determined that only the key gene STAT1 had a colocalization -positive locus with CHD, and STAT1 influenced CHD through this locus. Subsequently, a regulatory network was formed by 26 kyoto encyclopedia of genes and genomes pathways, 57 targets enriched by STAT1, and 51 active ingredients. Among them, the key gene STAT1 had a binding capacity of −7.0 kcal/mol with the active ingredient 6-Hydroxynaringenin. This study provides a scientific basis for the development of THSWD therapy for CHD by identifying a novel target (STAT1) and a compound (6-Hydroxynaringenin). These discoveries might offer novel objectives for diagnosis and treatment strategies of CHD.

## 1. Introduction

Coronary heart disease (CHD) is the leading cause of morbidity and mortality worldwide, and represents a worsening public health crisis with profound impacts on economic productivity. Its increasing prevalence highlights the need for comprehensive strategies that focus on prevention, early detection, and effective management.^[1]^ Recent advances in modern medicine have significantly improved CHD treatment outcomes, with drug therapy being the mainstay of clinical practice. However, conventional pharmacotherapies are limited by acquired drug resistance and the adverse effects associated with chronic administration. Therefore, safer and more effective treatments are urgently needed. A deeper understanding of the diagnosis, etiology, and pathogenesis of CHD, together with the identification of key genetic targets for drug treatment, holds promise for improving the prevention, diagnosis, and treatment of CHD.^[2,3]^

Taohong Siwu Decoction (THSWD), a classical prescription for promoting blood circulation and alleviating blood stasis, has attracted significant attention in both basic and clinical research. Recent studies have highlighted its efficacy in the prevention and treatment of cardiovascular diseases.^[4]^ Recent mechanistic studies indicate that THSWD protects against myocardial injury through multiple pathways, including suppression of inflammatory responses, enhancement of antioxidant activity, inhibition of platelet aggregation, prolongation of clotting time, attenuation of antifibrosis, reduction of lipid levels, promotion of anti-atherosclerotic activity, improvement in hemorheology, and modulation of vascular pathological changes. THSWD has also been shown to regulate various signaling pathways.^[5–8]^

Although THSWD has demonstrated therapeutic benefits in CHD treatment, the underlying mechanisms remain unclear and require further investigation. Mendelian randomization (MR), which uses genetic variants strongly associated with exposure factors as instrumental variables, offers a robust approach for evaluating causal relationships between exposures and outcomes.^[9]^ When integrated with network pharmacology, Mendelian randomization enables screening of disease-related biomarkers at various levels. Given that genetic variations are determined at birth and remain stable throughout life, they are unaffected by environmental factors. This characteristic allows them to serve as reliable instrumental variables linking exposure to outcomes and effectively addresses confounding biases, thereby enhancing the reliability and accuracy of the research findings.^[10,11]^

To systematically and comprehensively explore the potential mechanisms of THSWD in CHD treatment, we conducted an integrated analysis of its active components, its target genes, and CHD-related genes retrieved from various databases to identify THSWD-associated genes in CHD. Mendelian randomization analysis was performed to screen for candidate key genes, followed by colocalization analysis to confirm the final key genes. Further analyses, including the network construction of active components, disease pathways, and molecular docking between key genes and active compounds, were conducted. This study provides novel insights into the key genes and mechanisms underlying the therapeutic effects of THSWD in CHD.

## 2. Methods

### 2.1. Selection of drug active ingredients and drug-target genes

THSWD contains several traditional Chinese medicines (TCM), each of which contains potentially active ingredients. The potentially active ingredients of 6 traditional Chinese medicines (Honghua (*Carthami Flos*), Taoren (*Persicae Semen*), Danggui (*Angelicae Sinensis Radix*), Chuanxiong (*Chuanxiong Rhizoma*), Chishao (*Paeoniae Radix Rubra*), and Shudihuang (*Rehmanniae Radix Praeparata*)) were derived from the Traditional Chinese Medicine Systems Pharmacology Database and Analysis Platform (TCMSP) (https://tcmsp-e.com/tcmsp.php). The filtering criteria were oral bioavailability (OB) of at least 30% and drug-likeness (DL) of at least 0.18. For each drug, the top 10 active ingredients are listed in descending order of their OB values. If there were fewer than ten participants, all participants were listed. Additionally, the molecular formula of the active ingredient with the highest OB value is presented for each drug. Subsequently, Canonical SMILES of the active ingredients were obtained using PubChem (https://pubchem.ncbi.nlm.nih.gov/). Drug-target gene data were obtained from the SwissTargetPrediction database (http://swisstargetprediction.ch/). In this database, the Canonical SMILES of each active ingredient was entered separately, and the corresponding species “Homo sapiens” was selected to predict its associated targets. Target genes of the 6 active TCM ingredients were acquired after deduplication. Finally, 547 CHD-related genes (CHDRGs) were acquired from the Gene Cards database (https://www.genecards.org/) (Table S1, Supplemental Digital Content, https://links.lww.com/MD/R39).

### 2.2. Construction of the interaction network and identification of candidate genes

Based on the TCM components, active ingredients obtained in the previous step, and drug-target genes, the Cytoscape package (v 3.10.0)^[12]^ was used to create a TCM-active ingredient-drug-target interaction network. The gg Venn Diagram package (v 1.2.3)^[13]^ was used to determine the intersection of CHDRGs and target genes in order to identify candidate genes related to CHD treatment by THSWD.

### 2.3. Functional enrichment analysis of candidate genes

To gain an in-depth understanding of the biological functions and mechanisms of the candidate genes, we performed gene ontology (GO) and Kyoto encyclopedia of genes and genomes (KEGG) analyses using the cluster Profiler package (v 4.10.0).^[14]^ The screening criterion was set as an adjusted *P*-value (p.adj) <.05, with the Benjamini-Hochberg (BH) method applied for *P*-value correction. The GO analysis included biological processes (BP), molecular functions (MF), and cell components (CC). The enriched pathways were sorted from the smallest to the largest p.adj values. The GOplot package (v 1.0.2) was used to draw a Circos plot to display the genes enriched in the top 5 pathways of the 3 GO terms and the genes enriched in the top 5 KEGG pathways.^[15]^

### 2.4. Mendelian randomization analysis

The causal relationship between the candidate genes and CHD was further investigated. The CHD data (ieu-a-7) was obtained by using the Integrative Epidemiology Unit Open genome-wide association study (IEU Open GWAS) database (https://gwas.mrcieu.ac.uk/), including 184,305 samples (60,801 n case and 123,504 n control) and 9455,779 single nucleotide polymorphisms (SNPs). Expression quantitative trait loci (QTL) of the candidate genes were also obtained from the IEU Open GWAS database and utilized as exposure factors (Table S2, Supplemental Digital Content, https://links.lww.com/MD/R39).

The 3 core assumptions of Mendelian randomization included: The Relevance Assumption: that the genetic variant is significantly associated with the exposure; The Independence Assumption: that the genetic variant is independent of any confounders; The Exclusion Restriction Assumption: that the genetic variant affects the outcome only through the exposure, with no alternative pathways. To satisfy these 3 MR assumptions and identify key genes causally associated with CHD, we used the extract_instruments function from the TwoSample MR package (v 0.5.8) to extract exposures and screen for instrumental variables (IVs) with the following criteria: *P* < 5 × 10^−6^, *r*² = 0.001, kb = 10000. IVs that were significantly related to the exposure factors, but not to the outcomes, were selected using the extract outcome data function with the setting proxies TRUE and rsq = 0.8. Subsequently, the effect alleles and effect sizes were adjusted using the harmonized data function in the 2-sample MR, with the exposure factors and outcome data integrated to generate the MR analysis data.

MR analysis was then conducted using a combination of 6 algorithms MR-Egger,^[16]^ weighted median,^[17]^ inverse variance weighted (IVW),^[18]^ weighted mode,^[19]^ simple mode,^[20]^ and Wald ratio^[21]^ in which IVW was the most important analysis method (*P* < .05). An odds ratio (OR) > 1 was considered a risk factor, whereas an OR < 1 was regarded as a protective factor.

Following this, a scatter plot (a negative slope and a result of approximately 0 indicated that the gene was a protective gene for CHD and the study was not affected by confounders), a forest plot (genes with effect sizes to the left of the dashed line were considered protective genes, while on the opposite side was considered a risk factor), and a funnel plot (if the samples were symmetrically distributed around the IVW line, it implied that the MR complied with Mendel second law) were used to analyze the relationship between the exposure and outcome to further explain the analysis results of MR.

In addition, we performed a heterogeneity test using the mr heterogeneity function in the 2-sample MR, and Cochran Q statistic was calculated using IVW and MR-Egger regressions, which indicated that there was an absence of heterogeneity when *P* > .05. Otherwise, the IVW approach used random effects. Horizontal pleiotropy was examined by applying the Egger intercept within the 2-sample MR, which indicated no significant horizontal pleiotropy when *P* > .05. Leave-one-out (LOO) analysis was performed using the mr leave-one-out function to assess the robustness and reliability of MR results. To verify that the results of the MR analysis were not confounded by reverse causal effects, the Steiger test was performed (*P* < .05, correct causal direction = TRUE). Candidate genes causally associated with CHD are regarded as key candidate genes.

### 2.5. Bayesian test

A Bayesian test was performed to obtain the key genes between the expression quantitative trait loci (eQTLs) of candidate key genes and all SNPs within 500kb upstream and downstream of the leading SNP in CHD, using the Coloc package (v 5.2.3). Subsequently, the posterior probability (PP) of the shared variant tool was calculated. Colocalization analysis provided the following PPs to determine whether a solitary variant was common to 2 characteristics: PPH0, no association with either an eQTL or CHD. PPH1, a genetic variant associated only with eQTL but not with CHD. PPH2, a genetic variant associated with CHD but not with eQTL. PPH3 was associated with both eQTL and CHD with different causal variants. PPH4, a common causal variant associated with eQTL and CHD. PP.H4.abf > 0.8 was the applicable critical threshold for colocalization evidence of GWAS and eQTL association.

### 2.6. Construction of the active ingredient-target-pathway network

In order to understand the regulatory mechanism of key genes at the molecular level, a network of active ingredient – target – pathway was established using the Cytoscape package (v 3.10.0) based on the pathways enriched by key genes, the pathways combined with targets, and the active ingredients corresponding to the targets.

### 2.7. Molecular docking

To explore the correlation between key genes and the corresponding active ingredients of THSWD, 3-dimensional structures of proteins (key genes) were retrieved from the Protein Data Bank (PDB) database (http://www.rcsb.org). Subsequently, we removed the inherent small molecules and water molecules using PyMol software (v 2.5). Protein hydrogenation and charge calculations were performed using AutoDock Tools (v. 4.2.6).^[22]^ The 2D configurations of the active elements were retrieved from the PubChem database (https://pubchem.ncbi.nlm.nih.gov/), and charge balancing and rotatable bond checking of the small-molecule structures were performed using Auto Dock Tools (v 4.2.6). Finally, molecular docking was carried out using Auto Dock Vina to predict the binding affinity, and visualization was carried out using Py Mol software (v 2.5).^[23]^ A molecular docking binding affinity of less than – 5 kcal/mol indicates a stronger compound binding activity.

### 2.8. Statistical analysis

R (v. 4.2.2) was used for statistical analysis. Statistical significance was set at *P* < .05.

## 3. Results

### 3.1. Identification of 117 candidate genes

**Figure 1.**
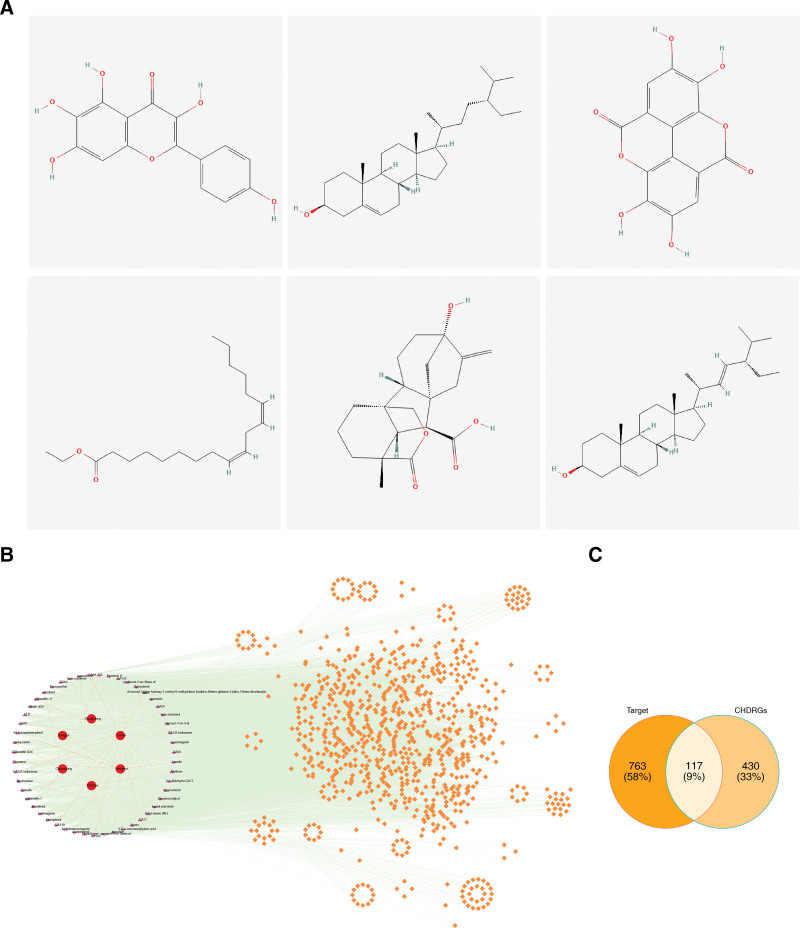
(A) The active ingredients with the highest OB values corresponding to the 6 constituent drugs were 6-Hydroxykaempferol, ellagic acid, ethyl linoleate, gibberellin A44, sitosterol, and beta-sitosterol. (B) The TCM-active ingredient-drug-target interaction network revealed 5119 interactions among 6 TCMs, 51 active ingredients, and 871 drug-target genes. Notably, Taoren (Prunus persica), Honghua (Carthamus tinctorius), and Chishao (Paeonia lactiflora) exhibited closer connectivity with these active ingredients, suggesting their key roles in the formula. (C) A total of 117 candidate genes were identified from the intersection of 880 drug target genes (derived from 83 active ingredients) and 547 CHDRGs. These genes were selected for subsequent functional enrichment and causal association analyses. CHDRGs = coronary heart disease-related genes, OB = oral bioavailability, TCMs = traditional Chinese medicines

After employing the databases, 83 active ingredients (OB > 30%, DL > 0.18) were predicted from the TCMSP using THSWD. The active ingredients with the highest OB values corresponding to the 6 drugs were 6-Hydroxykaempferol, ellagic acid, ethyl linoleate, gibberellin A44, sitosterol, and beta-sitosterol (Fig. 1A). The intersections of the drug targets corresponding to the 83 active ingredients were determined, and 880 drug-target genes were obtained (Table S3, Supplemental Digital Content, https://links.lww.com/MD/R39). The TCM-active ingredient-drug-target interaction network showed 5119 interaction relationships among the 6 TCMs, 51 active ingredients, and 871 drug-target genes (Fig. 1B) (Table S4, Supplemental Digital Content, https://links.lww.com/MD/R39). Taoren, Honghua, and Chi Shao have close connections to these active ingredients. Finally, 117 candidate genes were obtained from the intersections of 880 drug-target genes and 547 CHDRGs, which were used for subsequent functional enrichment analysis (Fig. 1C).

### 3.2. Enrichment analysis in 117 candidate genes

**Figure 2.**
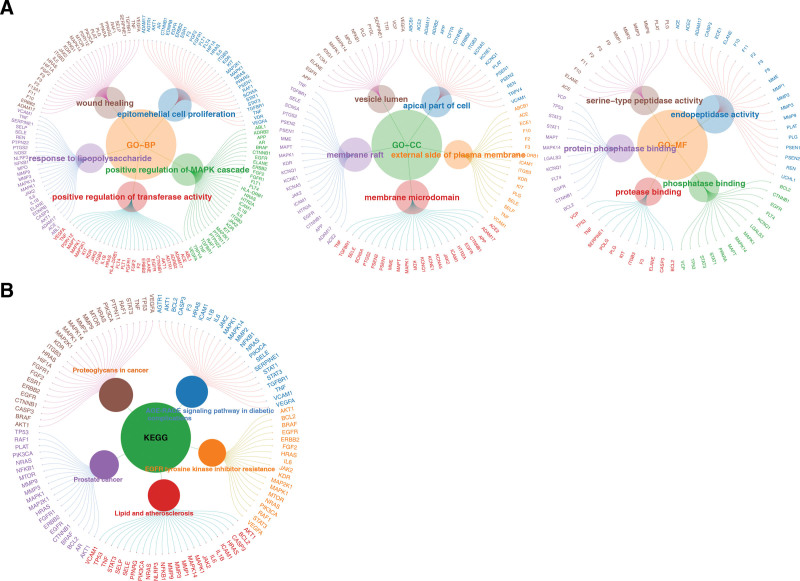
(A) Top 5 most significantly enriched GO BPs for the 117 candidate genes (adjusted *P*-value <.05). The enriched terms included epithelial cell proliferation, wound healing, positive regulation of the MAPK cascade, positive regulation of transferase activity, and response to lipopolysaccharide – all of which are functionally relevant to CHD pathophysiology. (B) Top 5 most significantly enriched KEGG pathways for the 117 candidate genes, along with their corresponding genes. The pathways included the AGE-RAGE signaling pathway in diabetic complications, proteoglycans in cancer, prostate cancer, EGFR tyrosine kinase inhibitor resistance, and lipid and atherosclerosis – highlighting the multi-pathway mechanisms underlying CHD. AGE-RAGE = advanced glycation end-products receptor for advanced glycation end-products, BPs = biological processes, CHD = coronary heart disease, EGFR = epidermal growth factor receptor, GO = gene ontology, KEGG = Kyoto encyclopedia of genes and genomes, MAPK = mitogen-activated protein kinase.

In total, 117 candidate genes were enriched in 2407 biological functions and pathways, and 2185 biological processes (BPs), 89 cellular components (CCs), and 133 molecular functions (MFs) were abundant (*P* < .05) (Table S5, Supplemental Digital Content, https://links.lww.com/MD/R39). Among the GO terms, the top 5 pathways with the most significant enrichment were epithelial cell proliferation, wound healing, positive regulation of the Mitogen-Activated Protein Kinase (MAPK) cascade, positive regulation of transferase activity, and response to lipopolysaccharide (Fig. 2A). In addition, 155 KEGG pathways were enhanced by 117 candidate genes (Table S6, Supplemental Digital Content, https://links.lww.com/MD/R39). The top 5 KEGG enrichment results and their corresponding genes are presented, including the advanced glycation end-product receptor for advanced glycation end-products (AGE-RAGE) signaling pathway in diabetic complications, proteoglycans in cancer, prostate cancer, epidermal growth factor receptor (EGFR) tyrosine kinase inhibitor resistance, and lipid and atherosclerosis (Fig. 2B).

### 3.3. MR analysis yields 5 candidate key genes

To identify candidate key genes causally associated with CHD, we performed MR analysis. First, through the screening of IVs and the harmonization of effect alleles and effect sizes, a total of 637 instrumental variables (IVs) corresponding to 91 candidate genes were finally retained, and 515 instrumental variables (IVs) remained after removing duplicates (Table S7, Supplemental Digital Content, https://links.lww.com/MD/R39), MR analysis was then performed. In IVW algorithm, after removing confounding factors, 5 candidate genes were significantly causally associated with CHD, where qtl-a-ENSG00000141736 (ERBB2) (OR = 1.081, 95% confidence interval (CI) = 1.000–1.169, *P* = .049), eqtl-a-ENSG00000134242 (PTPN22) (OR = 1.104, 95% CI = 1.049–1.162, *P* < .001), eqtl-a-ENSG00000102755 (FLT1) (OR = 1.087, 95% CI = 1.024–1.155, *P* = .006), eqtl-a-ENSG00000005381 (Myeloperoxidase, MPO) (OR = 1.046, 95% CI = 1.009–1.085, *P* = .014), and eqtl-a-ENSG00000115415 (STAT1) (OR = 1.148, 95% CI = 1.041–1.265, *P* = .006), all were risk factors (Table 1).

**Table 1:** Causal association results between 5 candidate genes.

3	eqtl-a-ENSG00000005381	ieu-a-7	Coronary heart disease ‖ id:ieu-a-7	‖ id:eqtl-a-ENSG00000005381	Inverse variance weighted	14	0.0454016417734529	0.0184840027674528	0.0140388897737885	0.00917299634924538	0.0816302871976605	1.04644807277532	1.0092151972177	1.08505457709529
91	eqtl-a-ENSG00000102755	ieu-a-7	Coronary heart disease ‖ id:ieu-a-7	‖ id:eqtl-a-ENSG00000102755	Inverse variance weighted	6	0.0838009572086193	0.0307366807671383	0.00640263797376626	0.0235570629050282	0.14404485151221	1.08741243066125	1.02383672217741	1.15493590798524
143	eqtl-a-ENSG00000115415	ieu-a-7	Coronary heart disease ‖ id:ieu-a-7	‖ id:eqtl-a-ENSG00000115415	Inverse variance weighted	19	0.137761999358538	0.0497760777838089	0.00564647089702025	0.040200886902273	0.235323111814804	1.14770236386739	1.04101988044732	1.26531754173686
186	eqtl-a-ENSG00000134242	ieu-a-7	Coronary heart disease ‖ id:ieu-a-7	‖ id:eqtl-a-ENSG00000134242	Inverse variance weighted	4	0.098806423484014	0.0260490316440641	0.000148781851836167	0.0477503214616483	0.14986252550638	1.10385259893557	1.04890873262837	1.16167453113248
204	eqtl-a-ENSG00000141736	ieu-a-7	Coronary heart disease ‖ id:ieu-a-7	‖ id:eqtl-a-ENSG00000141736	Inverse variance weighted	11	0.0781323449772102	0.0397677401187744	0.0494470306116062	0.000187574344412411	0.156077115610008	1.08126574929216	1.00018759193758	1.168916341

(ERBB2, PTPN22, FLT1, MPO, STAT1) and coronary heart disease (CHD) using the inverse variance weighting (IVW) algorithm. The table includes odds ratios (OR) (measure of effect size), 95% confidence intervals (CI) (precision of estimates), and p-values (statistical significance). All genes were classified as risk factors for CHD (e.g., STAT1: OR = 1.148, 95% CI = 1.041–1.265, *P* = .006).

Eqtl = expression quantitative trait loci, ERBB2 = Erb-b2 receptor tyrosine kinase 2

**Figure 3.**
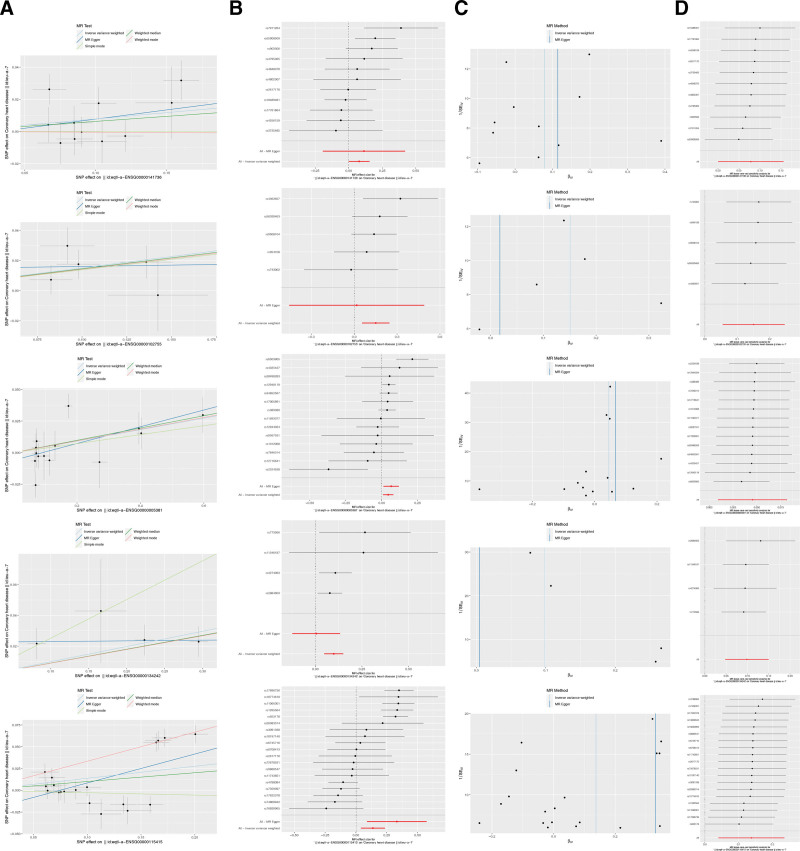
(A and B) Scatter plots (A) and forest plots (B) validating the causal associations between 5 candidate genes (ERBB2, PTPN22, FLT1, MPO, STAT1) and CHD. Using the IVW algorithm and adjusting for confounding factors, all 5 genes were identified as risk factors for CHD (e.g., ERBB2: OR = 1.081, 95% CI = 1.000–1.169, P = .049; STAT1: OR = 1.148, 95% CI = 1.041–1.265, P = .006). (C) Funnel plot demonstrating the symmetric distribution of IVs associated with the 5 candidate genes. This symmetry indicates adherence to Mendel second law and supports the reliability of the MR analysis. (D) Results of the LOO test for the 5 candidate genes. No abnormal SNPs were detected, confirming the stability and robustness of the MR results – i.e., no single SNP drove the observed causal associations. CHD = coronary heart disease, CI = confidence interval, ERBB2 = Erb-b2 receptor tyrosine kinase 2, IVs = instrumental variables, IVW = inverse variance weighting, LOO = leave-one-out, MR = Mendelian randomization, SNP = single nucleotide polymorphisms.

In addition, the results of the scatter and forest plots further confirmed that qtl-a-ENSG00000141736 (ERBB2), eqtl-a-ENSG00000134242 (PTPN22), eqtl-a-ENSG00000102755 (FLT1), eqtl-a-ENSG00000005381 (MPO), and eqtl-a-ENSG00000115415 (STAT1) acted as risk genes, but the influence of confounding factors was not significant (Fig. 3A and B). In the funnel plot, the SNPs associated with the candidate genes were found to be largely symmetrically distributed, suggesting that the MR analysis adhered to Mendel second law (Fig. 3C).

Sensitivity analyses were performed using multiple tests, and the test for heterogeneity showed that the effects of the 4 candidate genes on CHD showed no heterogeneity, whereas eqtl-a-ASG00000115415 (signal transducer and activator of transcription 1 [STAT1]) had a *P*-value of 0.000, and the results were still satisfactory using the IVW test for random effects (Table 2). Horizontal pleiotropy showed p-values of > 0.05 for 5 candidate genes, indicating that the 5 candidate genes passed the horizontal pleiotropy test (Table 3). We found no abnormal SNPs in the 5 candidate genes in the LOO test results (Fig. 3D), indicating that the MR results were reliable and stable. Finally, Steiger test verified the main direction between the 5 candidate genes and CHD, and the correct causal direction for the 5 candidate genes was TRUE (*P* < .05) (Table 4). Five candidate genes, ERBB2, PTPN22, FLT1, MPO, and STAT1, were identified as potential key genes for further analysis.

**Table 2:** Heterogeneity test results for the 5 candidate genes in MR analysis.

	id.exposure	id.outcome	outcome	exposure	method	Q	Q_df	Q_pval
1	eqtl-a-ENSG00000005381	ieu-a-7	Coronary heart disease ‖ id:ieu-a-7	‖ id:eqtl-a-ENSG00000005381	MR-Egger	19.685142645215	12	0.0732813765495021
2	eqtl-a-ENSG00000005381	ieu-a-7	Coronary heart disease ‖ id:ieu-a-7	‖ id:eqtl-a-ENSG00000005381	Inverse variance weighted	21.4359694139234	13	0.0647412487150455
3	eqtl-a-ENSG00000102755	ieu-a-7	Coronary heart disease ‖ id:ieu-a-7	‖ id:eqtl-a-ENSG00000102755	MR-Egger	2.79965917696934	3	0.423556025667928
4	eqtl-a-ENSG00000102755	ieu-a-7	Coronary heart disease ‖ id:ieu-a-7	‖ id:eqtl-a-ENSG00000102755	Inverse variance weighted	3.11741399846991	4	0.538372302126434
5	eqtl-a-ENSG00000115415	ieu-a-7	Coronary heart disease ‖ id:ieu-a-7	‖ id:eqtl-a-ENSG00000115415	MR-Egger	86.1537926368535	17	3.03562481543813e-11
6	eqtl-a-ENSG00000115415	ieu-a-7	Coronary heart disease ‖ id:ieu-a-7	‖ id:eqtl-a-ENSG00000115415	Inverse variance weighted	100.823290170275	18	1.56474850989007e-13
7	eqtl-a-ENSG00000134242	ieu-a-7	Coronary heart disease ‖ id:ieu-a-7	‖ id:eqtl-a-ENSG00000134242	MR-Egger	0.361942498112043	2	0.834459349844923
8	eqtl-a-ENSG00000134242	ieu-a-7	Coronary heart disease ‖ id:ieu-a-7	‖ id:eqtl-a-ENSG00000134242	Inverse variance weighted	2.83374725613886	3	0.41797460610755
9	eqtl-a-ENSG00000141736	ieu-a-7	Coronary heart disease ‖ id:ieu-a-7	‖ id:eqtl-a-ENSG00000141736	MR-Egger	13.7502034708533	9	0.131492550212762
10	eqtl-a-ENSG00000141736	ieu-a-7	Coronary heart disease ‖ id:ieu-a-7	‖ id:eqtl-a-ENSG00000141736	Inverse variance weighted	13.8363591449403	10	0.180587483858965

Four genes (ERBB2, PTPN22, FLT1, MPO) showed no significant heterogeneity (*P* > .05), supporting the use of fixed-effects IVW. For STAT1, significant heterogeneity was detected (*P* = .000), but the random-effects IVW model still yielded robust results, confirming the stability of the causal association.

Eqtl = expression quantitative trait loci, MR = Mendelian randomization, ERBB2 = Erb-b2 receptor tyrosine kinase 2.

**Table 3:** Horizontal pleiotropy test results (using the MR-Egger intercept) for the 5 candidate genes.

	id.exposure	id.outcome	outcome	exposure	egger_intercept	se	pval
1	eqtl-a-ENSG00000005381	ieu-a-7	Coronary heart disease ‖ id:ieu-a-7	‖ id:eqtl-a-ENSG00000005381	-0.006044784	0.00585110417965508	0.321928760347138
2	eqtl-a-ENSG00000102755	ieu-a-7	Coronary heart disease ‖ id:ieu-a-7	‖ id:eqtl-a-ENSG00000102755	0.0142648585833155	0.0253058771872492	0.612348993474989
3	eqtl-a-ENSG00000115415	ieu-a-7	Coronary heart disease ‖ id:ieu-a-7	‖ id:eqtl-a-ENSG00000115415	-0.024748744	0.0145464924305161	0.107093207650501
4	eqtl-a-ENSG00000134242	ieu-a-7	Coronary heart disease ‖ id:ieu-a-7	‖ id:eqtl-a-ENSG00000134242	0.0225267937735687	0.0143282219826178	0.256526206010051
5	eqtl-a-ENSG00000141736	ieu-a-7	Coronary heart disease ‖ id:ieu-a-7	‖ id:eqtl-a-ENSG00000141736	-0.003682411	0.0155068495890493	0.817609835295075

Note: All p-values were > 0.05, indicating no significant horizontal pleiotropy – i.e., the IVs (SNPs) associated with the genes did not affect CHD through alternative pathways, satisfying a key assumption of MR analysis.

Eqtl = expression quantitative trait loci

**Table 4:** Steiger directionality test results validating the causal direction between the 5 candidate genes and CHD.

id.exposure	id.outcome	exposure	outcome	snp_r2.exposure	snp_r2.outcome	correct_causal_direction	steiger_pval
eqtl-a-ENSG00000005381	ieu-a-7	‖ id:eqtl-a-ENSG00000005381	Coronary heart disease ‖ id:ieu-a-7	9.505 × 10^−02^	1.703 × 10^−04^	TRUE	0
eqtl-a-ENSG00000102755	ieu-a-7	‖ id:eqtl-a-ENSG00000102755	Coronary heart disease ‖ id:ieu-a-7	5.352 × 10^−02^	8.004 × 10^−05^	TRUE	6.70 × 10^−150^
eqtl-a-ENSG00000115415	ieu-a-7	‖ id:eqtl-a-ENSG00000115415	Coronary heart disease ‖ id:ieu-a-7	5.341 × 10^−02^	7.798 × 10^−04^	TRUE	1.21 × 10^−249^
eqtl-a-ENSG00000134242	ieu-a-7	‖ id:eqtl-a-ENSG00000134242	Coronary heart disease ‖ id:ieu-a-7	3.064 × 10^−02^	9.344 × 10^−05^	TRUE	1.99 × 10^−161^
eqtl-a-ENSG00000141736	ieu-a-7	‖ id:eqtl-a-ENSG00000141736	Coronary heart disease ‖ id:ieu-a-7	2.152 × 10^−02^	1.041 × 10^−04^	TRUE	2.88 × 10^−108^

All results were TRUE (correct causal direction) with *P* < .05, ruling out reverse causality (e.g., CHD affecting gene expression) and confirming that the genes drive CHD risk.

Eqtl = expression quantitative trait loci

### 3.4. Regulatory relationships between the key gene STAT1 and other molecules

**Figure 4.**
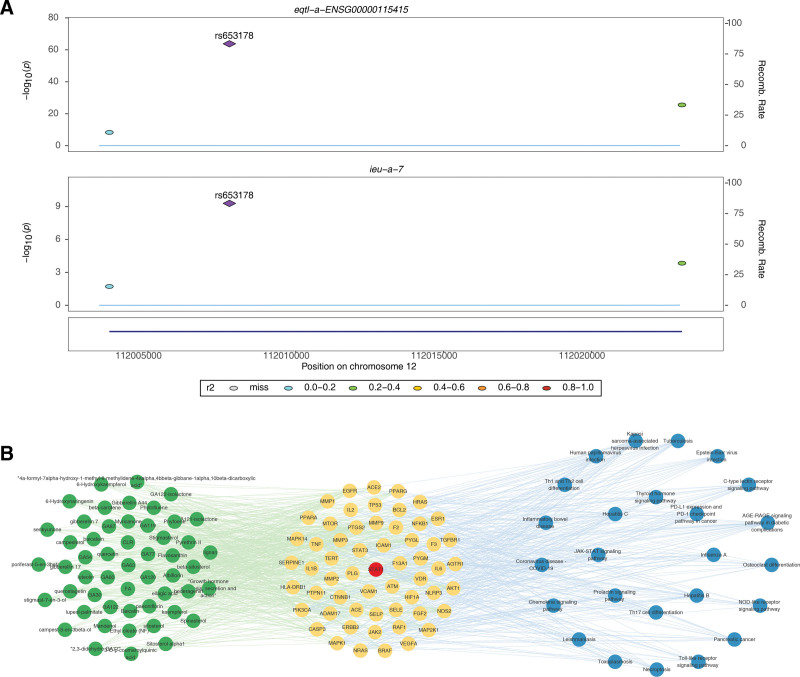
(A) Colocalization analysis of the STAT1 gene regon (chromosome 12: 111977756–112037756) with CHD. A pleiotropic SNP (rs653178) was identified as a shared causal variant, with a posterior probability of 99.99% (PP.H4.abf > 80%) for mediating the STAT1-CHD association. (B) The active ingredient-target-pathway network consisted of 888 interactions among 135 nodes (51 active ingredients, 57 targets, 26 KEGG pathways). STAT1 was enriched in 26 pathways (e.g., AGE-RAGE signaling, lipid metabolism) and targeted by 51 active ingredients, underscoring its central role in the formula’s anti-CHD mechanism. AGE-RAGE = advanced glycation end-products receptor for advanced glycation end-products, CHD = coronary heart disease, KEGG = kyoto encyclopedia of genes and genomes.

The results of the Bayesian test showed that among the 5 candidate key genes, only STAT1 (eqtl-a-ENSG00000115415) had a colocalization relationship with CHD (Table 5). There was only 1 colocalization-positive locus (PP.H4.abf > 80%), indicating a 99.99% probability of driving the phenotype. There was a pleiotropic SNP (rs653178) in the selected gene region, 12:111977756 to 112037756, for both STAT1 and CHD, suggesting that this gene influences the disease through this locus (Fig. 4A). STAT1 is a key gene in CHD. The active ingredient-target-pathway network consisted of 888 interrelationships between the 135 nodes. STAT1 was enriched in 26 KEGG pathways and targeted 57 targets related to 51 active components (Fig. 4B).

**Table 5:** Bayesian colocalization analysis results for the 5 candidate genes.

id.exposure	outcome	nsnp	t(chrpos)	nsnps	PP.H0.abf	PP.H1.abf	PP.H2.abf	PP.H3.abf	PP.H4.abf
eqtl-a-ENSG00000005381	Coronary heart disease ‖ id:ieu-a-7	14	17:56,343,613–56,403,613	161	6.04659223948713 × 10^−288^	0.934094866278846	3.0816197633311 × 10^−290^	0.00469936859931532	0.0612057651218809
eqtl-a-ENSG00000102755	Coronary heart disease ‖ id:ieu-a-7	6	13:28,999,431–29,059,431	178	2.92845439933325 × 10^−106^	0.167122867420038	1.45310619932215 × 10^−105^	0.829264122050829	0.0036130105291235
eqtl-a-ENSG00000115415	Coronary heart disease ‖ id:ieu-a-7	19	12:111977756–112037756	3	8.01040842702301 × 10^-64^	8.35568452222688 × 10^-07^	9.58682698912968 × 10^-61^	6.05509185037098 × 10^-09^	0.999999158376467
eqtl-a-ENSG00000134242	Coronary heart disease ‖ id:ieu-a-7	4	1:114,397,450–114,457,450	103	3.23575090729625 × 10^−123^	0.927776279907454	1.90848362760039 × 10^−125^	0.00540531431088307	0.0668184057816494
eqtl-a-ENSG00000141736	Coronary heart disease ‖ id:ieu-a-7	11	19:16,412,019–16,472,019	7	1.48456317284771 × 10^−30^	0.857728837613835	3.87936686453833 × 10^−34^	8.194707556986 × 10^−05^	0.142189215310602

Only STAT1 showed a significant colocalization with CHD (PP.H4.abf > 80%), indicating a shared causal variant between the gene and the disease. This further strengthened STAT1’s role as a key driver of CHD.

Eqtl = expression quantitative trait loci.

### 3.5. Strong binding energy level between 6-hydroxynaringenin and STAT1

**Figure 5.**
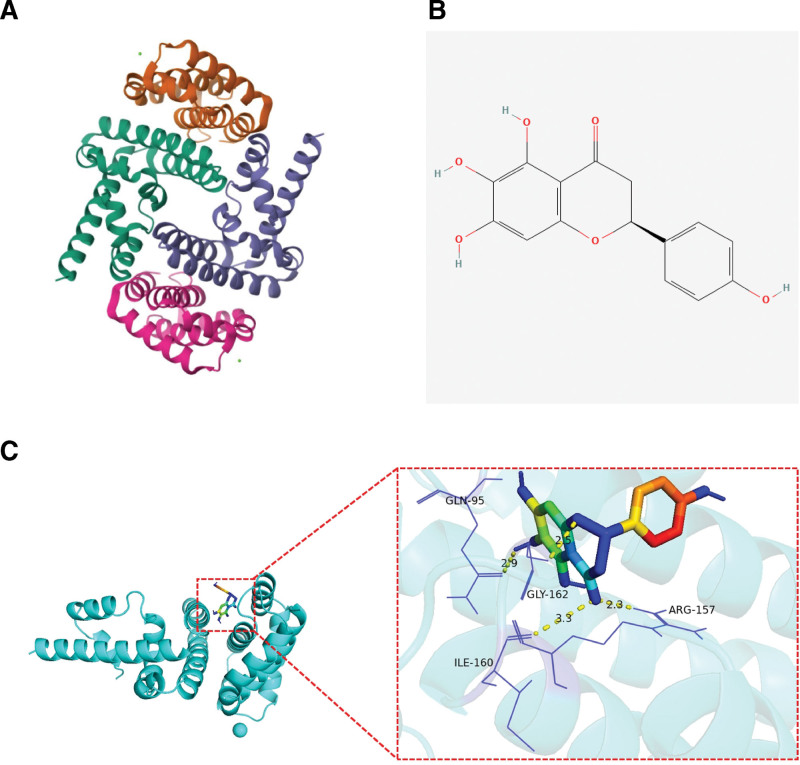
(A) 2-dimensional (2D) chemical structure of 6-Hydroxynaringenin obtained from the PubChem Database. (B) These structures provided the basis for molecular docking analysis. (C) Molecular docking results showing a binding free energy of −7.0 kcal/mol between STAT1 and 6-Hydroxynaringenin, indicating high-affinity binding. Hydrogen-bond interactions were observed between the ligand and 4 key amino acid residues (GLN-95, GLY-162, ARG-157, ILE-160) in the STAT1 active site, further validating their functional interaction.

The conformation of STAT1 was obtained from the PDB database, and the 2D structure of 6-Hydroxynaringenin was retrieved from the PubChem database (Fig. 5A and B). Through calculation, the free energy of binding between STAT1 and 6-Hydroxynaringenin was −7.0 kcal/mol, the binding activity between the 2 compounds was excellent, and there were hydrogen-bond interactions between the amino acid residues GLN-95, GLY-162, ARG-157, and ILE-160 and the 6-Hydroxynaringenin molecule (Fig. 5C).

## 4. Discussion

CHD, which is characterized by atherosclerotic plaque formation, vascular inflammation, and endothelial dysfunction^[24,25]^ and ultimately leads to myocardial ischemia or infarction, is the leading cause of mortality worldwide.^[26]^ Despite advances in modern therapeutics such as statins and antiplatelet agents, the high prevalence of residual risks and adverse effects underscores the need for alternative treatment strategies for CHD.

The therapeutic potential of Traditional Chinese medicine (TCM), which promotes blood circulation and resolves stasis, has been increasingly recognized in the management of CHD. Previous studies have shown that THSWD, a classic TCM formula composed of Taoren (*Persicae Semen*), Honghua (*Carthami Flos*), and Chishao (*Paeoniae Radix Rubra*),^[27]^ exerts protective effects against CHD by modulating lipid metabolism and reducing oxidative stress.^[7]^ However, the molecular mechanisms underlying its efficacy, particularly the key active ingredients, targets, and pathways involved remain poorly understood.^[28]^

In this study, we employed an integrated approach that combined network pharmacology, Mendelian randomization (MR), and molecular docking to systematically explore the therapeutic mechanisms of THSWD in CHD, thereby providing a scientific foundation for its clinical application. A total of 83 active ingredients in THSWD were identified using the TCMSP. Among these, Taoren, Honghua, and Chishao were the most closely associated with these active ingredients. From the intersection of 880 drug-target genes and 547 CHDRGs, 117 candidate genes were identified and subjected to functional enrichment analysis. Additionally, 155 KEGG pathways were enhanced by 117 candidate genes.

The identification of bioactive components with high OB and DL values is a critical step in elucidating the mechanisms of action of TCM formulations. Using the TCMSP database, we screened 83 active THSWD ingredients that met the criteria OB > 30% and DL > 0.18. Key compounds included 6-Hydroxykaempferol, ellagic acid, and beta-sitosterol, all of which are known for their potent antioxidant, anti-inflammatory, and vaso-protective properties, which play key roles in CHD pathogenesis. 6-Hydroxykaempferol, a flavonoid with the highest OB value, inhibits low-density lipoprotein (LDL) oxidation and reduces foam cell formation in atherosclerotic plaques.^[29]^ Ellagic acid, another major component, modulates NO production in endothelial cells and improves vascular relaxation.^[30]^ Notably, β-sitosterol, a plant-derived sterol, lowers serum cholesterol levels by competing for cholesterol absorption in the intestine, which is a critical factor in plaque progression.^[31]^ Taken together, these findings support our hypothesis that THSWD exerts therapeutic effects through multiple bioactive components that target the CHD-related pathways.

A key strength of our study was the integration of MR and Bayesian colocalization analyses to validate the causal relationships between candidate genes and CHD. Using MR, we identified 5 risk genes (ERBB2, PTPN22, FLT1, MPO, and STAT1) that may contribute to CHD susceptibility. Among these, only STAT1 showed a significant colocalization signal with CHD (PP.H4.abf > 80%), indicating a shared genetic locus (rs653178) driving both STAT1 expression and CHD risk.

This finding is particularly compelling because STAT1, a transcription factor, plays a pivotal role in regulating pro-inflammatory cytokines (e.g., tumor Necrosis Factor-α, Interleukin-6) and immune cell activation – processes that drive atherosclerotic plaque instability. Our results align with those of previous studies showing that STAT1 overexpression in muscle cells promotes plaque progression, whereas its inhibition reduces inflammation and improves endothelial function.^[32]^

STAT1 is located on chromosome 2 and comprises 24 exons, encoding a protein with a molecular weight of approximately 91–92kDa.^[33]^ As a key regulator of immune and inflammatory signaling, STAT1 is activated by cytokines, such as interferon (IFN). Activated STAT1 enters the nucleus and regulates the expression of several inflammation-related genes related to inflammation.^[34]^ STAT1 promotes the recruitment of immune cells to damaged coronary artery walls, intensifying the inflammatory response and thereby influencing CHD progression.^[32,35]^ Additionally, STAT1 modulates the function of immune cells such as macrophages and T lymphocytes, and their abnormal activation is closely associated with CHD development. Abnormal activation of STAT1 can lead to overproduction of inflammatory mediators and adhesion molecules by vascular endothelial cells, causing endothelial cell damage.^[36]^ One study demonstrated that inhibition of STAT1 activation reduced the production of pro-inflammatory cytokines, thereby alleviating vascular inflammation and improving endothelial function. These findings provide a theoretical basis for exploring STAT1 as a therapeutic target in CHD.

The active ingredient-target-pathway network constructed in this study revealed a complex interplay between 51 active ingredients, 57 targets, and 26 KEGG pathways, with STAT1 serving as the central node. This network highlights the “multi-component, multi-target, multi-pathway” characteristic of THSWD, a hallmark of TCM therapy. For example, 6-Hydroxynaringenin, a key active ingredient with high OB and DL values, was found to interact with STAT1 and 25 other targets, including genes involved in oxidative stress (e.g., SOD1) and lipid metabolism (e.g., LPL).^[37]^ Molecular docking further confirmed a strong binding affinity between 6-Hydroxynaringenin and STAT1 (-7.0 kcal/mol), with hydrogen bonds formed at residues Glutamine-95 (GLN-95), Glycine-162 (GLY-162), Arginine-157 (ARG-157), and Isoleucine-160 (ILE-160). These interactions suggest that 6-Hydroxynaringenin may inhibit STAT1-mediated transcription of pro-inflammatory genes, thereby attenuating vascular inflammation, a critical step in CHD progression.

Enrichment analysis of the STAT1-centered network identified several pathways relevant to CHD, including the advanced glycation end-product receptor for advanced glycation end-products (AGE-RAGE) signaling pathway,^[38]^ Mitogen-Activated Protein Kinase(MAPK) cascade, and Nuclear Factor kappa B (NF-κB) pathway. For example, the AGE-RAGE pathway is activated in patients with diabetes and CHD and contributes to endothelial dysfunction by increasing reactive oxygen species (ROS) production. Our findings suggest that THSWD modulates this pathway by inhibiting STAT1, thereby reducing oxidative stress and improving vascular integrity.^[39]^ Similarly, the MAPK cascade, which regulates cell proliferation and inflammation, is a known target of TCM components.^[40]^

Constructing an active component-target-pathway network can provide a more comprehensive understanding of the complex interactions among the genes involved in CHD development. In this network, the nodes represent genes or proteins, whereas the edges indicate regulatory relationships between them. Our analysis revealed that multiple STAT1-related genes form an intricate network, suggesting that targeting STAT1 might trigger a cascade of effects, thereby effectively regulating multiple pathogenic processes in CHD. Furthermore, certain natural compounds can activate or inhibit the activity of specific genes in this network, thereby offering potential directions for the development of novel drugs to treat CHD.

## 5. Implications and Limitations

This study provides novel insights into the therapeutic mechanism of THSWD in CHD, identifying STAT1 as a key target and 6-Hydroxynaringenin as a potential bioactive component. The integration of MR and network pharmacology addresses the limitations of traditional observational studies, thereby strengthening the causal inference between the THSWD components and CHD risk. However, several limitations should be noted: network pharmacology analysis relies on public databases, which may contain incomplete or biased data; MR analysis is limited by the availability of valid instrumental variables (IVs) and potential pleiotropy, although our sensitivity analyses (e.g., Steiger test, LOO) supported the reliability of our results; and molecular docking is a computational method that requires experimental validation (e.g., in vitro binding assays and animal studies) to confirm the functional relevance of the interactions observed.

## 6. Conclusion

Our study systematically elucidated the therapeutic mechanism of THSWD in CHD using a multiomics approach. We identified STAT1 as a key causal target and 6-Hydroxynaringenin as a potential active component with interactions mediated through pathways regulating inflammation, oxidative stress, and vascular function. These findings not only provide a scientific basis for the clinical use of THSWD in CHD, but also offer novel targets (e.g., STAT1) and compounds (e.g., 6-Hydroxynaringenin) for future drug development. Further experimental and clinical studies are warranted to validate these results and to translate them into therapeutic applications.

## Acknowledgments

Special recognition was accorded to the collaborators at the Xin Li research units for their technical guidance and constructive intellectual interaction. We gratefully acknowledge the researchers who made their data publicly available in the GEO database and recognized their significant contributions to advancing scientific inquiry through open data sharing. Finally, We gratefully acknowledge the funding support from the Sichuan Medical Association and the Sichuan Science and Technology Program, and declare no competing interests related to this work.

## Author contributions

**Conceptualization:** Chunbin Wang, Xin Li, Jian Wang.

**Data curation:** Chunbin Wang, Xin Li, Jian Wang.

**Formal analysis:** Chunbin Wang.

**Funding acquisition:** Chunbin Wang.

**Project administration:** Chunbin Wang.

**Software:** Chunbin Wang, Jian Wang.

**Supervision:** Jian Wang.

**Validation:** Chunbin Wang.

**Visualization:** Chunbin Wang, Xin Li, Jian Wang.

**Writing – original draft:** Chunbin Wang.

**Writing – review & editing:** Xin Li.

## Supplementary Material


